# Efeito Antioxidante e Anti-inflamatório do Suco de Laranja

**DOI:** 10.36660/abc.20210418

**Published:** 2021-06-08

**Authors:** Paula Felippe Martinez, Marianna Rabelo de Carvalho, Maria Lua Marques Mendonça, Marina Politi Okoshi, Silvio Assis de Oliveira-Junior

**Affiliations:** 1 Universidade Federal de Mato Grosso do Sul Campo GrandeMS Brasil Universidade Federal de Mato Grosso do Sul (UFMS), Campo Grande , MS - Brasil; 2 Departamento de Clínica Médica Faculdade de Medicina de Botucatu Universidade Estadual Paulista Júlio de Mesquita Filho BotucatuSP Brasil Departamento de Clínica Médica , Faculdade de Medicina de Botucatu , Universidade Estadual Paulista Júlio de Mesquita Filho (UNESP), Botucatu , SP - Brasil

**Keywords:** Suco de Frutas e Vegetais, Citrus Sinensis, Estresse Oxidativo, Metabolismo, Anti-inflamatórios, Remodelação Ventricular, Antioxidantes

A progressão da remodelação cardíaca após o infarto do miocárdio é um evento complexo que envolve diversas reações biológicas, dentre elas, estresse oxidativo e resposta inflamatória. ^1 - 4^ A liberação de espécies reativas de oxigênio (EROs), após injúria isquêmica do miocárdio, estimula o aumento de mediadores pró-inflamatórios, envolvidos na proliferação de fibroblastos e no reparo tecidual na região infartada. No entanto, a produção sustentada de EROs, associada com sobrecarga hemodinâmica e incapacidade do sistema antioxidante, induz estresse oxidativo em áreas não infartadas que também sofrem remodelação cardíaca. ^1 - 3^ Essas alterações, acompanhadas da ocorrência de desordens do metabolismo energético, ativação de metaloproteinases, morte e hipertrofia cardiomiocitária, fibrose intersticial e disfunção ventricular, caracterizam o processo de remodelação cardíaca. ^1^

Estudos sobre variados compostos bioativos, frequentemente extraídos de alimentos, têm sido conduzidos com o propósito de atenuar desordens comuns à remodelação cardíaca, como estresse oxidativo e inflamação. ^4 - 6^ Entretanto, a extração e isolamento dessas substâncias pode demandar complexa rede de procedimentos técnicos especializados e de alto custo, dificultando financeiramente o acesso de grande parte da população a esse recurso. Ademais, sua ingestão isolada não permite a avaliação de potenciais efeitos derivados da interação entre componentes da matriz alimentar e do organismo que possam alterar os efeitos metabólicos. ^7^ Por outro lado, o consumo do alimento em sua totalidade atua como tampão no sistema digestivo, o que permite maior biodisponibilidade de seus compostos ativos. Em vista disso, abordagens nutricionais envolvendo ingestão de alimentos naturais, como frutas e seus produtos (cascas, sucos, polpas, purê, geleia, entre outros), que tenham em sua composição substâncias com propriedades antioxidantes e anti-inflamatórias, têm despertado cada vez mais interesse. ^6 , 8^ Nesse contexto, cabe destaque às frutas cítricas, ricas em compostos bioativos que podem promover alterações metabólicas e proteger tecidos de lesões resultantes do acúmulo de EROs. ^7^ Sucos de frutas cítricas, em geral, são fontes abundantes de vitamina C e contribuem com o fornecimento de outros nutrientes, como potássio, folato, magnésio, além de vitamina A ^9^ e compostos polifenólicos. ^10^ Nesse sentido, o suco de laranja é uma matriz alimentar complexa e tem demonstrado potencial cardioprotetor devido à sua composição e capacidade antioxidante e anti-inflamatória. ^9 - 11^

Na presente edição dos Arquivos Brasileiros de Cardiologia, Oliveira et al. ^12^ mostram os benefícios do consumo de suco de laranja, levando-se em conta seu papel antioxidante e cardioprotetor, em modelo experimental de remodelação cardíaca após infarto do miocárdio. Conforme esperado, o infarto de miocárdio desencadeou processo de remodelação cardíaca, caracterizada por hipertrofia cardíaca e prejuízo do desempenho sistólico e diastólico do ventrículo esquerdo, acompanhados de aumento do estresse oxidativo e de marcadores inflamatórios e alterações do metabolismo energético. O consumo de suco de laranja melhorou a função sistólica e diastólica do ventrículo esquerdo, assim como diminuiu a atividade da glutationa peroxidase e a concentração de interferon gama (INF-γ) no miocárdio de animais infartados. Com relação ao metabolismo energético, os ratos infartados que consumiram suco de laranja mostraram maior atividade de ATP sintase e fosfofrutoquinase, enzimas chave do metabolismo energético. Outro achado importante desse estudo foi o aumento da expressão proteica da heme-oxigenase-1, sugerindo importante efeito antioxidante e anti-inflamatório em resposta ao tratamento com suco de laranja em animais infartados.

Portanto, há indícios de que a inclusão de produtos naturais na dieta pode contribuir como adjuvante na atenuação da remodelação cardíaca pós infarto do miocárdio. Estudos futuros são necessários para melhor elucidar os efeitos cardioprotetores de intervenções com este e outros produtos naturais.
