# Supplementary material for: Efeito Antioxidante e Anti-inflamatório do Suco de Laranja
Source: Arq Bras Cardiol. 2021 Jun 8;116(6):1137–8. [Article in Portuguese] doi: 10.36660/abc.20210418 (PMC8288538; doi:10.36660/abc.20210418)
Supplement: Translation

# Translation: Antioxidant and Anti-Inflammatory Effects of Orange Juice

**Authors:** Paula Felippe Martinez, Marianna Rabelo de Carvalho, Maria Lua Marques Mendonça, Marina Politi Okoshi, Silvio Assis de Oliveira-Junior

**Affiliations:** 1 Brasil Universidade Federal de Mato Grosso do Sul (UFMS), Campo Grande, MS - Brasil; 2 Brasil Departamento de Clínica Médica, Faculdade de Medicina de Botucatu, Universidade Estadual Paulista Júlio de Mesquita Filho (UNESP), Botucatu, SP - Brasil

**Keywords:** Fruit and Vegetable Juices, Citrus Sinensis, Oxidative Stress, Metabolism, Anti-Inflammatory Agents, Ventricular Remodeling, Antioxidants

Post myocardial infarction cardiac remodelling progression is a complex event involving several biological reactions; these include oxidative stress and inflammatory response.^1 - 4^ The release of reactive oxygen species (ROS) after myocardial ischemia stimulates pro-inflammatory mediators involved in fibroblast proliferation and tissue repair in the infarcted area. However, sustained ROS production in combination with hemodynamic overload and antioxidant system incapacity leads to oxidative stress in non-infarcted areas, which also undergo cardiac remodelling.^1 - 3^ These changes, associated with energy metabolism disorders, metalloproteinases activation, cardiomyocyte death and hypertrophy, interstitial fibrosis, and cardiac dysfunction characterize the cardiac remodelling process.^1^

Studies on various bioactive compounds, often extracted from food, have been undertaken to assess their attenuating powers on disorders common to cardiac remodelling, such as oxidative stress and inflammation.^4 - 6^ However, the extraction and isolation of these substances can require a complex network of costly specialized technical procedures making it financially difficult for a large part of the population to access this resource. Additionally, taken in isolation one cannot evaluate the potential effects of interactions between food matrix components and the organism which may alter the metabolic effects.^7^ Alternatively, consuming the food in its entirety acts as a buffer in the digestive system allowing greater bioavailability of its active compounds. Therefore, nutritional approaches involving ingestion of natural foods, such as fruits and their products (peels, juices, pulps, puree, and jams, etc), which contain substances with antioxidant and anti-inflammatory properties, have increasingly gained interest.^6 , 8^ It is worth mentioning citrus fruits, which are rich in bioactive compounds that may change the metabolism and protect tissues from ROS accumulation injuries.^7^ Citrus fruit juices, in general, are abundant sources of vitamin C and other nutrients, such as potassium, folate, magnesium, vitamin A,^9^ and polyphenolic compounds.^10^ In this sense, orange juice is a complex food matrix with cardioprotective potential due to its antioxidant and anti-inflammatory capacity.^9 - 11^

In this edition of ABC, Oliveira et al.^12^ show the benefits of orange juice consumption, taking into account its antioxidant and cardioprotective role, in rats with cardiac remodelling after myocardial infarction. As expected, myocardial infarction triggered the cardiac remodelling process, characterized by cardiac hypertrophy and impaired left ventricle systolic and diastolic performance, accompanied by increased oxidative stress and inflammatory markers, and changed energy metabolism. Consumption of orange juice improved left ventricle systolic and diastolic dysfunction, and decreased myocardial glutathione peroxidase activity and interferon gamma (INF-γ) concentration. Regarding energetic metabolism, infarcted rats that consumed orange juice had higher myocardial ATP synthase and phosphofructokinase activity, key enzymes of energy metabolism. Another important finding in the study was increased heme-oxigenase-1 expression, suggesting an antioxidant and anti-inflammatory response to treatment with orange juice in infarcted animals.

There is therefore evidence that including natural products in the diet contributes as an adjunct in attenuating cardiac remodelling after myocardial infarction. Future studies are needed to better elucidate the cardioprotective effects of interventions with this and other natural products.
